# Depression, higher level of tension induction, and impaired coping strategies in response to stress in women with PCOS correlate with clinical and laboratory indices of hyperandrogenism and not with central obesity and insulin resistance

**DOI:** 10.1007/s00737-024-01500-x

**Published:** 2024-08-17

**Authors:** Edyta Dutkiewicz, Dominik Rachoń, Miłosz Dziedziak, Agnieszka Kowalewska, Joanna Moryś

**Affiliations:** 1 Department of Clinical and Experimental Endocrinology, Medical University of Gdańsk, Dębinki 7, 80-211 Gdańsk, Poland; 2 Private Outpatient Clinic, Słonimskiego 8/50, 80-280 Gdańsk, Poland; 3 Department of Clinical Psychology, Medical University of Gdańsk, Tuwima 15, 80-210 Gdańsk, Poland

**Keywords:** Personality, Self-esteem, Mood, Emotions, Anxiety

## Abstract

**Abstract:**

PCOS is characterized by ovarian hyperandrogenism and insulin resistance (IR), which give rise to symptoms of hyperandrogenism and central obesity, which in turn may cause depression, lower self-esteem, and deteriorate coping strategies in stressful situations.

**The purpose:**

to examine the mental condition, self-esteem, and ways of coping with stress in women with PCOS compared to age and BMI-matched healthy controls and to correlate them with clinical and laboratory hyperandrogenism, central obesity, and IR.

**Methods:**

42 women with PCOS and 39 controls were assessed for the above-mentioned psychological measures and correlated with serum hormonal and metabolic parameters.

**Results:**

Compared to controls, women with PCOS had more symptoms of depression (p = 0.026), a higher level of tension induction (p = 0.032), were more prone to alcohol consumption (p = 0.015), and were less likely to use the strategy of active coping in stressful situations (p = 0.014) and to seek instrumental (p = 0.048) and emotional support (p = 0.043). The presence of hirsutism correlated negatively with the level of emotional induction (R = -0.32, p < 0.05), and androgenic alopecia positively with the hedonistic tone (R = 0.36, p < 0.05). Serum testosterone (TST) correlated positively with the likelihood of seeking instrumental support in stressful situations (R = 0.31, p < 0.05) and with emotional focus (R = 0.34, p < 0.05). Serum androstenedione (A4-dione) correlated negatively with the escape behavior (R = -0.32, p < 0.05). No correlations were found between waist circumference and IR with the studied psychological measures.

**Conclusions:**

Women with PCOS are characterized by depression, higher levels of tension induction, and impaired coping strategies in stressful situations, which correlate with clinical and laboratory indices of hyperandrogenism and not with central obesity and IR.

## 1. Introduction

Polycystic Ovary Syndrome (PCOS) is a heterogenous endocrine disorder characterized by hyperandrogenism of ovarian origin and intrinsic insulin resistance (IR) (Azziz et al. 2016; Diamanti-Kandarakis & Dunaif 2012). Depending on the diagnostic criteria used, PCOS affects from 5 to 20% of women of reproductive age (Broekmans & Fauser 2006; Yildiz et al. 2012). Excess ovarian androgen production in women with PCOS not only leads to menstrual disturbances (amenorrhea or oligomenorrhea) and fertility problems but also gives rise to signs of hyperandrogenism such as hirsutism, androgenetic alopecia and acne (Pasquali et al. 2016). Due to the intrinsic IR with its consequent hyperinsulinemia, women with PCOS develop central obesity and its adverse metabolic consequences, such as type 2 diabetes mellitus (T2DM), atherogenic dyslipidaemia, and arterial hypertension (Rachoń & Teede 2010). All these metabolic derangements in turn increase the risk of developing coronary heart disease, which still remains the leading cause of death in women worldwide (Lim et al. 2019; Osibogun 2020). It can be assumed that all the above-mentioned symptoms and consequences, regardless of their severity, may significantly affect the well-being of women affected by this syndrome (Cooney et al. 2017). This may result in a deterioration of mood, self-esteem (Joshi et al. 2022), and satisfaction with life (Rzońca et al. 2018), as well as the emergence of negative emotions such as anger and anxiety (Akdağ Cirik et al. 2016; Dokras 2012). However, studies on factors responsible for the deterioration of these psychological health measures in women with PCOS are lacking. Taking into account the psychological aspects of this syndrome (anxiety-depressive disorders), the early psychological diagnosis would, therefore, accelerate the involvement of psychotherapeutic interventions to improve mood, self-esteem, and quality of life. This, in turn, would facilitate the implementation of non-pharmacological treatment strategies for PCOS, which remain the first line of treatment, such as lifestyle change comprising the improvement of eating habits and the increase in physical activity (Dutkowska et al. 2019).

Therefore, our study was aimed at examining the mental condition, personality traits, self-esteem, and ways of coping with stress in women with PCOS compared to age and BMI-matched healthy controls and to correlate them with indices of clinical and laboratory hyperandrogenism, central obesity, and IR.

## 2. Methods

### Study subjects

The study was conducted among 42 women with PCOS (age range 19–42 yrs) and 39 control subjects (age range 18–49 yrs), who were recruited from two outpatient endocrinological practices based in the Pomeranian region of Poland (one in Gdansk and other in Gdynia) by a specialist in internal medicine and endocrinology (D.R.). The diagnosis of PCOS was made according to the AE&PCOS criteria, which include the presence of clinical or biochemical hyperandrogenism and ovarian dysfunction defined as anovulation or oligomenorrhea or/and the presence of polycystic ovary morphology (PCOM) on transvaginal ultrasound (TV USG) (Azziz et al. 2006). Control subjects were healthy women who were referred to the same outpatient endocrinological practices to exclude some of the common endocrine disorders (i.e. thyroid dysfunction). They had regular menses and no signs of clinical or laboratory hyperandrogenism. An interview-based medical form was used to obtain information regarding gynaecological and obstetric history (menarche, menstrual regularity, number of pregnancies and oral contraceptive use). The following strategies were applied to control for the potential biases: rigorous criteria for participant selection and assignment were used; participants were from the same general population; interactions between study subjects were with only one researcher; out-of-date control parameters were not used; the studies were registered with the appropriate bodies; a search was done if similar studies have already occurred. Women who were pregnant or were current users of oral contraceptives, glucocorticosteroids or anti-androgens (i.e. cyproterone acetate or spironolactone) as well as any anti-depressants and mood stabilizers were not included in the study. The height, body weight, waist circumference and blood pressure were measured as well as the presence of hirsutism, androgenic alopecia or acne were recorded. Participants were then referred to a specialist in gynaecology and endocrinology (A.K.) who performed gynaecological examination, together with a TV or transabdominal (TA) USG, which was performed using the the Accuvix V20 Ultrasound Machine (Medison, Seoul, South Korea), 4–9 MHz and 4–8 MHz, respectively. The ovarian volume was calculated and the total number of antral follicles was counted. PCOM was defined as an antral follicle count (AFC) of ≥ 12 in 2–9 mm diameter and/or ovarian volume of ≥ 10 ml at least in one ovary (Dewailly et al. 2013).

The study protocol was approved by the Ethics Committee of the Medical University of Gdańsk (permission number NKBBN/412/2018) and all the study participants gave a written consent to participate.

### Laboratory measurements

After the recruitment process all the study participants were referred to a medical laboratory (ALAB-Bruss Laboratories, Gdynia, Poland) were total blood count and serum concentrations of selected hormonal and metabolic parameters were measured. Serum thyrotropin (TSH), luteinizing hormone (LH), follicle-stimulating hormone (FSH), prolactin (PRL), 17β-oestradiol (E2), dehydroepiandrosterone sulphate (DHEA-S), total testosterone (TST), serum sex hormone binding globulin (SHBG), glucose and insulin concentrations were measured in an electrochemiluminescence immunoassay using Roche Diagnostics-Cobas ECLIA system commercial kits. Serum androstenedione (A4-dione) concentrations were evaluated using the CLIA method on a Liason analyser (Diasorin, Saluggia, Italy). Free androgen index (FAI) was calculated according to the formula: FAI = TSTx100/SHGB. Insulin resistance (IR) was assessed using the Homeostasis Model of Assessment-Insulin Resistance (HOMA-IR) according to the formula: fasting insulin (μU/mL) x fasting glucose (mmol/L) / 22.5.

### Psychological measures

#### Depression

All the study subjects were assessed for mood disorders using the standardized Beck’s Depression Inventory II (BDI II). The questionnaire consists of 21 items, each with four possible responses graded from 0 to 3. The total score ranges from 0 to 63 points. The severity of depression symptoms was determined by summing the points obtained in Beck's Inventory. Scores between 0–11, 12–19, 20–25, and 26–63 indicated no depression, mild depression, moderate depression, and severe depression, respectively.

#### Self-esteem

The assessment of self-esteem was carried out using the Rosenberg Self-Esteem Scale (RSES). This scale consists of ten statements, each offering four response options on a four-point Likert-type scale (1 = completely agree to 4 = completely disagree). The RSES has two factors: the first factor includes affirmative statements (1, 2, 4, 6, 7), while the second factor comprises negative statements (3, 5, 8, 9, 10). A higher score on the scale indicates elevated levels of global self-esteem, with a potential point range of 10 to 40 points.

#### Personality

The revised NEO (Neuroticism-Extraversion-Openness) Five-Factor Inventory (NEO-FFI) was utilized to assess the study subject’s character on five dimensions of personality: Extraversion, Agreeableness, Openness, Conscientiousness, and Neuroticism (the so-called Big Five personality traits). It asks participants to rate their own self-descriptive statements on a five-point Likert-type scale from 1 (strongly disagree) to 5 (strongly agree). The results of the 12 items are added together to determine the scores for each domain. There are 28 NEO FFI items in all that are reverse-worded.

#### Coping strategies in response to stress

The Coping Orientation to Problems Experienced (COPE) Multidimensional Inventory was used to evaluate various coping mechanisms employed by the study subjects in response to stress. It comprises 60 items and 15 first-order factors, each with 4 items. The first-order factors are based on conceptualizations of functional coping and earlier findings that suggested facilitating and impeding variables for adaptive coping (Carver & Scheier 1981) and include: 1-Acceptance, 2-Active coping, 3-Behavioral disengagement, 4-Denial, 5-Seeking emotional support, 6-Humor, 7-Seeking instrumental support, 8-Mental disengagement/self-distraction, 9-Planning, 10-Positive reinterpretation, 11-Religion, 12-Restraint, 13-Substance use, 14-Suppression of competing activities, and 15.-Venting. The study participants were asked to rate how frequently they use various coping strategies on a four-point Likert-type scale from 1 (I usually do not do this at all) to 4 (“I usually do this a lot).

#### Mood

Polish adaptation of the University of Wales Institute of Science and Technology (UWIST) Mood Adjective Checklist (MACL) was used to measure mood states (Goryńska et al. 2011). Energetic (EA), Tense (TA), and Hedonic (HT) are the three subscales that make up the scale. A four-point Likert-type scale from 1 (definitely yes) to 4 (definitely no) was used by the study participants to rate how well each adjective described their current mood state.

### Statistical analyses

IBM SPSS Statistics 28 package was used to calculate the descriptive statistics and to verify the research hypotheses. The data are presented as mean (± SD) and median (min–max). Shapiro–Wilk test was used to check the distribution of the variables. Since the distribution of all the variables was skewed non-parametric tests were used. The U Mann–Whitney test was used to compare the data between the two studied groups. The Rank Spearman test was used to calculate the correlation coefficients between the studied variables. Cronbach’s alpha coefficient measures were used to evaluate the reliability of the set of survey items. Cronbach’s alpha coefficient > 0.70 indicated good internal validity and reliability of the research tools. P value < 0.05 was considered statistically significant.

## Results

### Study subjects’ characteristics and comparisons between the studied groups

Study subjects’ characteristics and comparisons between the studied groups are presented in Table 1. Women from the studied groups did not differ in age, BMI, and waist circumference. There were no significant differences between serum TSH, FSH, and E2 concentrations between women with PCOS and the controls (*p* = 0.857, *p* = 0.242, and *p* = 0.212, respectively). Serum concentrations of LH, PRL, DHEA-S, A4-dione, and TST were significantly higher in women with PCOS (*p* = 0.023, p = 0.008, *p* < 0.001, *p* = 0.002 and *p* = 0.006, respectively) whereas serum concentrations of SHBG were significantly lower compared to the control subjects (*p* = 0.010). Higher TST and lower SHBG concentrations in women with PCOS resulted in higher FAI values in this group compared to the controls (*P* = 0.010). There were no significant differences in serum fasting glucose, insulin, and HOMA-IR between the studied groups (*p* = 0.240, *p* = 0.106, and *p* = 0.115, respectively).

**Table 1:** Comparison of the studied anthropometric, hormonal, metabolic, and inflammatory parameters between women with PCOS (*n* = 42) and the control subjects (*n* = 39)

	PCOS (*n* = 42)					Controls (*n* = 39)					
	mean	SD	median	min	max	mean	SD	median	min	max	P value
Age (years)	27	6.3	25.5	19	42	27	7.8	23	18	49	0.866
BMI	27.20	5.31	25.975	19.2	43.8	25.41	6.33	22.4	17.2	37.62	0.170
Waist (cm)	90.36	15.38	89.5	65	150	84.69	16.98	79	62	118	0.119
TSH (mIU/l)	2.15	0.99	2.125	0.46	5.29	2.20	1.32	1.95	0.539	8.25	0.857
LH (mIU/ml)	8.17	2.16	8.17	4.16	14	7.11	1.89	7.1	2.9	11.42	0.023*
FSH (mIU/ml)	6.91	1.21	6.91	4.12	11.62	6.47	1.99	6.5	3.77	14.09	0.242
PRL (ng/ml)	315.13	175.55	308.1	14.91	724.9	211.13	169.58	243.7	9.3	619.3	0.008*
E2 (pmol/l)	223.72	120.37	223.7	70.45	820	278.21	243.05	245	73	1340	0.212
DHEA-s (μg/dl)	326.19	100.11	326.2	124	588.3	227.21	52.90	227.2	92.76	377.5	< 0.001*
A4-dione (ng/ml)	2.96	0.71	2.96	2.1	5.43	2.42	0.83	2.42	1.032	5.08	0.002*
TST (nmol/l)	1.68	0.56	1.61	0.5	3.15	1.38	0.36	1.34	0.76	2.11	0.006*
SHBG (nmol/l)	67.21	36.40	67.2	24.77	195.7	66.36	17.1	65.1	30.2	112.7	0.010*
FAI	3.07	1.82	2.67	0.31	11.13	2.22	0.90	2.09	0.90	5.80	0.010*
Glucose(mg/dl)	91.19	10.30	89	75	115	88.72	8.29	87	77	112	0.240
Insulin (μlU/ml)	10.74	4.12	10.4	2.12	26.13	9.29	3.79	8.34	3.2	21.3	0.106
HOMA-IR	2.45	1.14	2.28	0.39	7.23	2.07	1.00	1.77	0.65	5.89	0.115

*BMI *body mass index, *TSH *thyroid stimulating hormone, *LH *luteinizing hormone, *FSH *follicle stimulating hormone, *PRL *prolactin, *E2 *17β-estradiol, *DHEA-S *dehydroepiandrosterone sulphate, *A4-dione * androstenedione, *TST * total testosterone, *SHGB *sex hormone binding globulin, *FAI *free androgen index, *HOMA-IR *homeostasis Model of Assessment-Insulin Resistance

* *p* < 0.05

### Analysis of the reliability of questionnaires used for psychological measures

First, an analysis of the reliability of the questionnaires (research tools) used in the study was performed using the Cronbach’s alpha coefficient measures. The scale of active coping did not achieve satisfactory reliability, but after removing one of the items (question no. 47), its reliability turned out to be satisfactory (Cronbach’s alpha coefficient 0.7). The scale of distraction did not achieve satisfactory reliability (Cronbach’s alpha coefficient 0.12), but it was further analyzed due to its specificity – each item of the questionnaire focused on different areas. Therefore, the consistency of the answers was not obligatory. The exact values of Cronbach's alpha of all scales are presented in Table 2.

**Table 2:** Reliability of the research tools used (N – number of items)

	Cronbach’s alpha	N
1. Depression	0.89	21
2. Hedonistic tone	0.94	10
3. Emotional induction	0.88	9
4. Tensional induction	0.90	9
5. Self-esteem	0.91	10
6. Active coping	0.70	3
7. Planning	0.77	4
8. Searching for instrumental support	0.84	4
9. Seeking emotional support	0.92	4
10. Avoiding competing activities	0.71	4
11. Turn to religion	0.97	4
12. Positive re-evaluation	0.70	4
13. Refraining from action	0.61	4
14. Acceptance	0.77	4
15. Focus on emotions	0.76	4
16. Denial	0.73	4
17. A distraction	0.12	4
18. Cessation of activities	0.65	4
19. Alcohol or drug abuse	0.96	4
20. Sense of humor	0.82	4
21. Active coping – general indicator	0.82	5
22. Escape behavior – general indicator	0.63	6
23. Seeking support and focusing on emotions – general indicator	0.71	4
24. Conscientiousness	0.86	12
25. Extroversion	0.84	12
26. Amicability	0.73	12
27. Neuroticism	0.87	12
28. Openness	0.70	12

### Comparison of the studied psychological measures between women with PCOS and the control subjects

In the next step, a comparison between women with PCOS and the control subjects in terms of psychological measures was performed. Scales such as symptoms of depression, mood indicators, self-esteem, coping strategies, and the level of the “big five” personality traits were analyzed. The analysis showed that women with PCOS, compared to women from the control group, had statistically significant more (1) symptoms of depression (*p* = 0.026), a higher level of (2) tension induction (*p* = 0.032), were more often prone to (3) alcohol consumption in stressful situations (*p* = 0.015), and were less likely to use the (4) strategy of active coping in stressful situations (*p* = 0.014) and to seek (5) instrumental (*p* = 0.048) and (6) emotional support (*p* = 0.043). Differences in the case of the first four measures were moderate (η2 = 0.06, η2 = 0.06, η2 = 0.08, η2 = 0.08, respectively) and small for the remaining two (η2 = 0.05 for both). The results are presented in Table 3.

**Table 3:** Comparison of the studied psychological measures between women with PCOS (*n* = 42) and the control subjects (*n* = 39)

	PCOS (*n* = 42)		Controls (*n* = 39)		*P *value	η^2^
	Mean	SD	Mean	SD		
Depression	14.14	8.85	9.67	7.67	0.026*	0.06
Hedonistic tone	28.80	6.14	31.10	6.06	0.065	0.04
Emotional induction	25.34	4.95	26.97	5.14	0.146	0.03
Tensional induction	19.88	6.01	17.08	5.00	0.032*	0.06
self-esteem	29.31	6.54	28.79	5.19	0.726	0.00
Active coping	2.91	0.62	3.22	0.51	0.014*	0.08
Planning	2.83	0.62	3.00	0.60	0.192	0.02
Searching for instrumental support	2.80	0.70	3.10	0.70	0.048*	0.05
Seeking emotional support	2.70	0.85	3.08	0.79	0.043*	0.05
Avoiding competing activities	2.50	0.54	2.71	0.54	0.132	0.03
Turn to religion	1.77	1.06	1.69	0.88	0.834	0.00
Positive re-evaluation	2.75	0.59	2.88	0.56	0.251	0.02
Refraining from action	2.47	0.49	2.44	0.57	0.777	0.00
Acceptance	2.53	0.54	2.54	0.68	0.752	0.00
Focus on emotions	2.85	0.64	2.88	0.76	0.723	0.00
Denial	1.67	0.51	1.71	0.62	0.915	0.00
A distraction	2.27	0.43	2.22	0.54	0.884	0.00
Cessation of activities	1.91	0.56	1.68	0.50	0.087	0.04
Alcohol or drug abuse	1.70	0.82	1.29	0.49	0.015*	0.08
Sense of humor	1.62	0.55	1.80	0.64	0.239	0.02
Active coping – general indicator	2.69	0.45	2.85	0.40	0.194	0.02
Escape behavior – general indicator	1.95	0.35	1.87	0.34	0.345	0.01
Seeking support and focusing on emotions – general indicator	2.53	0.59	2.68	0.59	0.192	0.02
Conscientiousness	32.90	6.40	31.90	8.61	0.626	0.00
Extroversion	27.48	7.04	27.46	8.72	0.996	0.00
Amicability	32.05	5.48	31.62	5.90	0.809	0.00
Neuroticism	25.69	10.04	23.28	9.60	0.321	0.00
Openness	28.74	6.51	30.72	6.12	0.183	0.01

η^2^- effect size

* *p* < 0.05

### Correlations of psychological measures with clinical and laboratory findings in women with PCOS

In the next step, correlations between the studied psychological measures among women with PCOS, and the clinical (presence of hirsutism, alopecia, acne) or laboratory (serum TST, DHEA-S, A4-dione and FAI) indices of hyperandrogenism as well as BMI, waist circumference and insulin resistance (HOMA-IR) were analyzed. The presence of hirsutism correlated negatively with the level of emotional induction (R = -0.32, *p* < 0.05) and the presence of androgenic alopecia correlated positively with the hedonistic tone (R = 0.36, *p* < 0.05). No correlations were found between the presence of acne and the studied psychological measures. Serum TST correlated positively with the likelihood of seeking instrumental support in stressful situations (R = 0.31, *p* < 0.05) and with the emotional focus (R = 0.34, *p* < 0.05) whereas serum A4-dione correlated negatively with the escape behavior (R = -0.32, *p* < 0.05). No correlations were found between serum DHEA-S, FAI, BMI, waist circumference, HOMA-IR, and the studied psychological measures. The data are presented in Table 4.

**Table 4:** Correlations of psychological measures with the clinical and laboratory indices of hyperandrogenism, BMI, waist circumference, and HOMA-IR in women with PCOS

	Hirsutism	Alopecia	Acne	TST	DHEA-s	A4-dione	FAI	BMI	Waist	HOMA
Depression	0,03	-0,16	0,01	-0,05	0,05	-0,06	-0,11	0,19	0,10	0,03
Hedonistic tone	0,03	0,36*	0,14	0,00	0,07	0,16	0,05	0,02	0,06	0,12
Emotional induction	-0,31*	0,17	0,03	0,15	0,08	0,11	0,12	0,02	-0,03	0,16
Tensional induction	0,18	-0,14	-0,04	-0,02	-0,01	-0,10	-0,02	-0,05	0,01	-0,15
self-esteem	-0,06	0,14	0,05	0,01	-0,21	0,29^	-0,23	-0,15	-0,17	-0,01
Active coping	0,01	0,23	-0,01	0,29^	0,10	-0,08	0,04	-0,04	0,00	0,07
Planning	-0,06	0,00	-0,08	0,11	0,22	-0,08	-0,16	0,03	0,06	0,10
Searching for instrumental support	-0,03	-0,04	-0,07	0,31*	0,04	-0,15	-0,07	-0,19	-0,08	-0,07
Seeking emotional support	0,13	0,02	-0,04	0,23	-0,01	-0,16	-0,2	-0,15	-0,06	-0,18
Avoiding competing activities	-0,22	0,10	-0,17	0,07	-0,11	0,03	0,00	-0,24	-0,24	0,05
Turn to religion	-0,03	0,16	0,04	0,18	0,1	0,02	0,21	0,05	0,00	0,13
Positive re-evaluation	-0,21	0,13	-0,07	0,11	0,07	0,03	0,02	-0,11	-0,10	-0,03
Refraining from action	-0,08	0,03	-0,02	0,1	0,08	0,02	0,10	-0,08	-0,21	-0,01
Acceptance	-0,07	-0,11	0,16	0,18	0,28^	-0,19	0,20	-0,07	-0,14	-0,13
Focus on emotions	0,04	-0,11	-0,30^	0,34*	-0,07	0,02	0,08	0,01	0,06	0,13
Denial	-0,18	0,15	-0,01	-0,08	0,00	-0,30^	-0,04	0,01	0,03	-0,02
A distraction	0,15	0,07	-0,03	0,00	-0,03	0,06	0,07	0,17	0,29^	0,16
Cessation of activities	-0,04	-0,07	-0,13	-0,06	-0,10	-0,27^	0,08	0,00	0,02	-0,16
Alcohol or drug abuse	0,10	0,26	0,00	0,08	-0,14	-0,19	-0,14	-0,07	0,06	-0,18
Sense of humor	0,10	0,17	-0,06	0,06	-0,08	-0,09	0,22	-0,11	0,12	-0,07
Active coping – general indicator	-0,11	0,15	-0,05	0,19	0,02	-0,01	-0,03	-0,13	-0,13	0,01
Escape behavior – general indicator	0,06	0,15	-0,05	0,02	-0,04	-0,32*	0,08	-0,05	0,09	-0,13
Seeking support and focusing on emotions – general indicator	0,08	0,09	-0,05	0,23	0,04	-0,08	-0,06	-0,09	-0,03	0,00
Conscientiousness	-0,19	-0,04	-0,02	0,20	0,13	-0,10	-0,19	0,04	-0,12	-0,01
Extroversion	-0,03	-0,08	0,01	0,17	0,16	-0,06	0,07	0,10	0,19	0,16
Amicability	0,08	-0,05	0,24	0,09	0,14	-0,04	-0,01	-0,05	-0,06	-0,12
Neuroticism	0,09	-0,10	0,03	-0,11	0,08	-0,04	0,12	0,06	0,08	-0,15
Openness	0,06	0,26	-0,05	0,05	0,19	-0,01	0,10	0,00	0,17	0,03

*** *p* < 0.001; ** *p* < 0.01; * *p* < 0.05

^ 0.05 < *p* < 0.1

## Discussion

PCOS is the most common endocrinopathy in women. Apart from menstrual disturbances due to oligo- or anovulation which cause fertility problems, the hallmark of this syndrome comprises clinical or laboratory hyperandrogenism and IR which lead to the development of central obesity and its complications (Rachoń & Teede 2010). On the other hand, changes in the body appearance may affect the well-being of women affected by this syndrome and cause depression, lower self-esteem, and deteriorate coping strategies in stressful situations (Elsenbruch et al. 2006). All these psychological measures usually impair the course of non-pharmacological treatment strategies for this syndrome, which involve changes in lifestyle comprising improvement of eating habits and physical activity (Dutkowska et al. 2019). Therefore, this study aimed to evaluate personality traits, the scale of depression and mood, self-esteem as well as coping strategies in women with PCOS compared to age and BMI-matched controls, and to correlate them with indices of clinical and laboratory hyperandrogenism, central obesity, and IR.

The results of our study showed that women with PCOS had significantly higher serum levels of LH, PRL, DHEA-S, A4-dione, TST, and FAI compared to the control subjects, which in turn is the hallmark of PCOS diagnosis. Increased LH levels in women with PCOS stimulate the ovarian production of androgen precursors (DHEA-S and A4-dione) by the theca cells and therefore lead to increased serum TST levels and FAI giving rise to the symptoms of hyperandrogenism such as hirsutism, acne and androgenic alopecia (Rachoń, 2012). On the other hand, A4-dione is also a precursor of estrone which is converted to E2 by the 17β-hydroxysteroid dehydrogenase (17β-HSD). Therefore, women with PCOS are also characterized by relative hyperestrogenism, which in turn may explain higher PRL levels in these individuals (Barnes 1998). Nevertheless, serum E2 levels in women with PCOS in our study did not differ compared to the control subjects. However, studies of other authors point to the higher estrone to E2 ratio in women with PCOS (Kim & Chun 2021). Unfortunately, serum estrone concentrations were not measured in our subjects.

Many studies show that women with PCOS may be more prone to the development of depression and anxiety (Deeks et al. 2010; Teede et al. 2010). A comprehensive systematic review performed by Cooney et al. (2017), which included 30 cross-sectional studies, representing 3 050 subjects with PCOS and 3 858 controls, from 10 different countries showed that women with PCOS compared to BMI-matched control subjects had higher odds ratios of both depressive (OR: 3.25; 95% CI 1.73–6.09; four studies) and anxiety symptoms (OR: 6.30, 95% CI: 1.88–21.09; three studies). Women with PCOS and concurrent depression in this study were older and had higher mean values of BMI, hirsutism scores, and IR, while women with PCOS and concurrent anxiety had higher mean values of BMI, hirsutism score, and serum free testosterone concentrations. The prevalence of depression in women with PCOS is estimated to be as high as 36.6% (Teede et al. 2018). Data from a study conducted in Brazil by Rassi et al. (2010) among 72 PCOS women showed a high prevalence of mental disorders (57%). Among them, the most prevalent was major depression (26%). Mood disorders occurred in 78% of the subjects, and severe depression in 26%. However, another study conducted among Polish women found that 73% of women diagnosed with PCOS showed symptoms of depressive disorders (Pokora et al. 2022).

In our study, the prevalence of depressive symptoms among women with PCOS was 59%. However, we have also found that our PCOS subjects compared to the BMI and aged-matched controls were characterized by a higher level of tension induction, were more often prone to alcohol consumption, and were less likely to use the strategy of active coping in stressful situations as well as to seek instrumental and emotional support (escape behavior). Therefore, in the next step, we aimed to investigate which of the studied psychological measures in women with PCOS correlated with the clinical (presence of hirsutism, acne, or alopecia) or laboratory indices of hyperandrogenism (serum TST, DHEA-S, A4-dione, and FAI) as well as BMI, waist circumference and IR, which all comprise a hallmark of this syndrome. There was a significant negative correlation between the level of emotional induction and the presence of hirsutism and a positive correlation between the hedonistic tone and the presence of alopecia. No correlations were found between the studied psychological measures and the presence of acne. Kolahi et al. (2015) have also shown that the presence of acne in women with PCOS does not affect the quality of life and coping strategies. The results of our study have also pointed to a positive correlation between the likelihood of seeking instrumental support in stressful situations and serum TST concentrations. Serum TST levels were also positively correlated with the emotional focus whereas serum A4-dione concentrations correlated negatively with the escape behavior. No correlations were found between serum BMI, waist circumference, HOMA-IR, and the studied psychological measures.

Emotional induction and focus on emotions as well as the escape behavior in stressful situations are psychological measures characteristic of both anxiety and depression. Morshedi et al. (2021) have found a correlation between the quality of life in women with PCOS and emotional and problem-solving coping strategies. Alcohol consumption is an example of an escape behavior and PCOS women in our study were also more often prone to alcohol drinking. There are no other studies that evaluated the use of alcohol as a coping strategy in women with PCOS, however, depression and anxiety are risk factors for alcohol abuse (Kessler et al. 1997). Active coping strategies involve an awareness of the stressor, followed by attempts to reduce the negative outcome. They are characterized by solving problems, seeking information, social support, and professional help, planning activities, and redefining the meanings of problems (Dijkstra & Homan 2016). POCS women in our study were less likely to use the strategy of active coping in stressful situations. A study conducted in Pakistan also showed that women with PCOS use less active practical coping strategies and more active distractive coping strategies (Fatima et al. 2021). Instrumental support is positively associated with mental health while emotional support is positively associated with overall well-being (Sheikh et al. 2016). PCOS women in our study were less likely to seek instrumental and emotional support than the control subjects. What is more, the likelihood of seeking instrumental support in stressful situations was positively correlated with serum TST concentrations.

Studies on the relationships between serum levels of sex hormones and anxiety-depressive disorders comprise an important area in psycho-neuro-endocrinological research and may play an important role in the treatment strategies of women with PCOS. Women in general are more vulnerable to anxiety and stress-related disorders compared to men, which points to the role of sex hormones, particularly E2 and progesterone, as well as testosterone in the pathogenesis of these gender differences (Li & Graham 2017; McHenry et al. 2014).

Estrogen exposure has been shown to influence certain signaling processes involved in the pathogenesis of anxiety symptoms. However, the role of estrogens in the pathogenesis of anxiety symptoms seems to be very complex. Rats exposed to high estrogen concentrations are characterized by anxiolytic behavior by exhibiting different forms of anxiety that relate to the changes in the function of the serotonergic system (Pandaranandaka et al. 2009). In contrast, several studies have found a correlation between decreased serum estrogen levels, i.e. premenstrually, during the postpartum period, and perimenopausally, and increased anxiety and depressive symptoms (Osterlund et al. 2005). Estrogen replacement in turn has been shown to have beneficial effects in postmenopausal women with anxiety and depressive symptoms. Estrogens modify some neural functions in the hippocampus and amygdala, which play a key role in regulating the stress response system (Walf & Frye 2006). Data from recent studies also indicate that the estrogen receptor beta (ERβ) appears to be a major mediator of the antidepressant and anxiolytic effects of estrogens (Osterlund et al. 2005).

The role of progesterone in these processes also seems to be very multifaceted. Exposure to progesterone has been shown to have mood-stabilizing, anxiolytic, and sometimes antidepressant effects due to metabolic pathways involving the production of allopregnanolone or other metabolites that act via GABA receptors (Chen et al. 2021; Majewska 1992). In contrast, a rise in progesterone levels during the luteal phase in women has been shown to promote subjective anxiety (Reynolds et al. 2018) This can be due to the antiestrogenic effects of progesterone which can attenuate the psychotropic effect of E2 on the brain, thus reducing emotional well-being (Bitzer 2010).

TST being the main androgen in men also plays a great role in women’s mental health. TST exposure has been shown to have anxiolytic and antidepressant effects in both men and women (McHenry et al. 2014). Nevertheless, these effects also appear to be very complex (Tyagi et al. 2017). A meta-analysis conducted by Maharjan et al. (2021) showed that higher serum TST levels characterize premenopausal women with depression. However, after Mendelian randomization analysis, the directions of the effect of this relationship were conflicting and could also be due to menopausal status. Also, data from a study conducted by Stanikova et al. (2019) showed that premenopausal women with symptoms of depression had higher serum free TST concentrations compared to women without depressive symptomatology. The activational and organizational effects of TST seem to contribute to these outcomes, albeit the exact underlying processes are yet unknown (McHenry et al. 2014).

The complexity of these effects may also be augmented by the fact that exposure to the adrenal androgen precursor the DHEA(-S) has been shown to influence well-being. Increased serum DHEA-S concentrations in women have been associated with greater amount, frequency, and enjoyment of leisure activities and healthier psychological profiles (Maninger et al. 2009). In contrast, serum DHEA-S levels were found to be negatively correlated with ratings of depressed mood in elder women (Barrett-Connor et al. 1999). What is more, postmenopausal women with post-traumatic stress disorder (PTSD) were characterized by a greater DHEA increase after adrenal activation with a synthetic ACTH (1–24) compared to non-traumatized subjects (Rasmusson et al. 2004). Data from a small study conducted by Olff et al. (2007) have also shown that psychotherapy in PTSD patients leads to an increase in serum DHEA concentrations. Therefore, it has been postulated that an increase in serum DHEA(-S) observed in PTSD may play a role in resilience and successful adaptation to stress.

Repeated DHEA administration in bulbectomized mice ameliorates cognitive deficits and depressive-like behaviors by enhancing neurogenesis via activation of the sigma-1 receptor in the hippocampus (Moriguchi et al. 2013). In a double-blind controlled study, treatment of patients with major depression with DHEA was associated with a significantly greater decrease in the Hamilton depression scale compared to placebo (Wolkowitz et al. 1999). There are no data on the effects of A4-dione on mental health measures. However, since DHEA is a precursor of this steroid, we might speculate that they would be comparable or similar. Nevertheless, data from our study showed that hyperandrogenism in women with PCOS correlates negatively with signs of anxiety-depressive symptoms, which point to the complexity of the effects of sex hormones on mental health in women.

In summary, women with PCOS in our study were characterized by depression, higher levels of tension induction, and impaired coping strategies in stressful situations, which correlated with clinical and laboratory indices of hyperandrogenism and not with central obesity and IR.
